# A drug comorbidity index to predict mortality in men with castration resistant prostate cancer

**DOI:** 10.1371/journal.pone.0255239

**Published:** 2021-07-28

**Authors:** Giuseppe Fallara, Rolf Gedeborg, Anna Bill-Axelson, Hans Garmo, Pär Stattin

**Affiliations:** 1 Division of Experimental Oncology/Unit of Urology URI, IRCCS Ospedale San Raffaele, Milan, Italy; 2 Vita‐Salute San Raffaele University, Milan, Italy; 3 Department of Surgical Sciences, Uppsala University, Uppsala, Sweden; 4 Regional Cancer Centre, Uppsala/Örebro, Uppsala University Hospital, Uppsala, Sweden; Istituto Scientifico Romagnolo per lo Studio e la Cura dei Tumori (IRST) - IRCCS, ITALY

## Abstract

**Background:**

The Charlson Comorbidity Index is a poor predictor of mortality in men with castration resistant prostate cancer (CRPC). To improve this prediction, we created a comorbidity index based on filled prescriptions intended to be used in registry-based studies.

**Materials and methods:**

In a population-based cohort of men with CPRC a drug comorbidity index (DCI-CRPC) was calculated based on prescriptions filled during a 365-day period before the date of CRPC diagnosis to predict mortality. Five risk categories for men with CRPC were defined based on PSA kinetics. Mortality rates were described by Kaplan-Meier curves. The predictive ability of the DCI-CRPC was compared in univariable models to that of the original DCI, derived from men in the general population, and to that of the Charlson Comorbidity Index.

**Results:**

In 1,885 men with CRPC the median overall survival ranged from 3.0 years (95% confidence interval [CI] 2.8 to 3.4) in the first tertile of the DCI-CRPC, to 1.0 year (95% CI 0.9 to 1.1) in the third tertile of the DCI-CRPC. The index had higher discriminative ability (C-index 0.667) than the Charlson Comorbidity Index (C-index 0.508). The discriminative ability of the DCI-CRPC was highest in the subgroup with least aggressive cancer (C-index 0.651) and lowest in men with most aggressive cancer (C-index 0.618). The performance of the DCI-CRPC was comparable to that of the original DCI.

**Conclusion:**

Our newly created comorbidity index using filled prescriptions predicted death in men with CRPC better than the Charlson Comorbidity Index.

## Background

There is a wide range in survival for men with prostate cancer who progress to castration resistant prostate cancer (CRPC) [1]. Measures such as the Eastern Cooperative Oncology Group performance status can predict survival for men with CRPC but they are rarely available in administrative health care databases or clinical cancer registers. The Charlson Comorbidity Index (CCI) based on hospital discharge diagnoses in administrative registers is more widely available and is a good predictor of survival in the general population and men with localized prostate cancer but is a poor predictor of overall survival (OS) in men with CRPC [2–5].

Several models based on filled prescriptions for drugs have been developed to predict mortality in various settings [6–8]. Recently, we created and validated a Drug Comorbidity Index (original DCI) to predict death from any cause in a cohort of men randomly selected as prostate cancer-free controls to men with prostate cancer [9,10]. Our original DCI was based on fillings for 106 drugs in the Swedish Prescribed Drug Register, which were associated with the risk of death. Each drug was assigned a specific weight that reflected its univariable association with survival probability. These weights were used to calculate an index predicting the survival probability for each man based on his filled prescriptions during the preceding year. The DCI predicted survival well within strata of age and CCI, even in the highest age group. The discrimination of this DCI was higher compared to the Rx-Risk Comorbidity Index, another previously published prescription-based risk index [6].

The aim of the present study was to create a Drug Comorbidity Index for prediction of all-cause mortality in men with CRPC, which perform better than the Charlson Comorbidity Index (CCI). The DCI is intended to be used as a measure of baseline health status indicative of risk of death from all causes in register-based studies.

## Material and methods

### Data sources and study population

The Uppsala-Örebro PSA cohort (UPSAC) database contains all Prostate-Specific Antigen (PSA) measurements obtained between 2005 and 2014 in the five regions in the Uppsala health-care region in Sweden [11]. By use of the unique Swedish personal identity number [12] and exact person-based linkage the UPSAC was linked to the National Prostate Cancer Register (NPCR) [13], the Cause of Death Register [14], the Swedish Prescribed Drug Register [15], and the National Patient Register [16]. Criteria for inclusion in the study population of men with CRPC were: 1) registration in NPCR; 2) first treatment with gonadotropin releasing hormone (GnRH) agonist/antagonist after 1 January 2006; 3) a doubling of nadir PSA value to >2 ng/mL or an absolute increase of 5 ng/mL or more, while on androgen deprivation therapy with GnRH or bilateral orchidectomy. In addition, men included in the study population had to be on androgen deprivation therapy for at least 3 months within a 6-month period according to the Prescribed Drug Register. Start of follow up was the date of CRPC diagnosis according to this definition. End of follow-up was 31 December 2014.

### Covariates

Tumor Node Metastasis (TNM) stage [17], Gleason Grade Groups [18], and data on diagnostic work-up and treatment were retrieved from NPCR [19]. The Charlson Comorbidity Index (CCI) was computed based on hospital discharge diagnoses in the National Patient Register from the 10-year period preceding the start of follow-up [20]. The CCI component for metastatic disease was excluded from the index. The study population was stratified into five categories according to their expected mortality risk based on the PSA at CRPC diagnosis and the PSA doubling time, which are known to be predictive of the risk of death in this population [21]. Cause and date of death were retrieved by linkage to the Cause of Death Register [22].

### The Drug Comorbidity Index (DCI)

The DCI was computed from filled prescriptions in the Prescribed Drug Register [23]. Two DCIs were calculated: the original DCI, based on the 106 drugs and their weights from our previous study on men in the general population [9,10], and a new CRPC-specific index (DCI-CRPC), calculated based on weights derived in the present study population of men with CRPC, using the same method as described for the original DCI [10].

### Statistical methods

We selected drugs for which a prescription had been filled by at least 1% of the men who died within the 365-day period preceding the start of follow-up (**S1 Table**) [10]. Each drug was identified with the anatomical therapeutic chemical classification (ATC) code at the chemical subgroup level. For each ATC code, a Cox univariable model was fitted to obtain the hazard ratio for death from any cause for men who had filled at least one prescription for that drug. The DCI-CRPC was calculated for each subject by adding the logarithm of the estimated ATC code specific hazard ratios (logHRs) corresponding to the subject’s filled prescriptions. The original DCI was calculated in the same way but using the previously published logHRs derived from men in the general population [10].

The discriminative ability of the DCI-CRPC and original DCI was assessed by fitting univariable Cox regression models and compare Harrell’s C-indices. Kaplan-Meier (KM) curves of overall survival were plotted stratified for tertiles of the DCI, for all men and for the five CRPC risk categories separately.

To penalize for internal validation, C-indices were calculated after bootstrapping (1000 samples) parameter estimates. Calibration curves at 1, 2, and 5 years of follow-up were plotted. Finally, the C-indices for DCI-CRPC, the original DCI, and CCI were compared.

Sensitivity analyses were performed by 1) using a more detailed ATC code level (the pharmacological subgroup level); 2) reducing the time period before the date of start of follow-up from which the filled prescriptions were retrieved, from 365 days to 180 days; 3) limiting the analysis to drugs that were prescribed to at least 5% of men who died; 4) including only ATC codes with parameter estimate p values was ≤ 0.2 from the Wald-test in a Cox regression model.

The study was approved by the Research Ethics Review Board in Uppsala that waived the informed consent requirement.

## Results

The study included 1885 men with CRPC, equally distributed between CRPC risk categories (**Table 1**). The higher the risk category, the higher the proportion of men with high PSA, advanced TNM stage, and high Gleason at the time of prostate cancer diagnosis. The median follow-up time of men in the study was 3.7 (interquartile range 1.2–4.4) years.

**Table 1:** Baseline characteristics of men with castration-resistant prostate cancer (CRPC) in the Uppsala-Örebro PSA cohort (UPSAC) database.

		All	CRPC low-risk category	CRPC low to mid-risk category	CRPC intermediate-risk category	CRPC mid to high-risk category	CRPC high-risk category
		(n = 1885)	(n = 355)	(n = 394)	(n = 361)	(n = 391)	(n = 384)
Clinical tumour (T) stage, n (%)	T1	269 (14)	54 (15)	53 (14)	65 (18)	45 (12)	52 (14)
	T2	469 (25)	104 (29)	98 (25)	81 (22)	103 (26)	83 (22)
	T3	893 (47)	167 (47)	195 (50)	167 (46)	185 (47)	179 (47)
	T4	221 (12)	24 (7)	39 (10)	43 (12)	52 (13)	63 (16)
	Missing	33 (2)	6 (2)	9 (2)	5 (1)	6 (2)	7 (2)
Node (N) stage, n (%)	N0	125 (7)	17 (5)	22 (6)	25 (7)	34 (9)	27 (7)
	N1	93 (5)	11 (3)	17 (4)	16 (4)	29 (7)	20 (5)
	NX	1667 (88)	327 (92)	355 (90)	320 (89)	328 (84)	337 (88)
Metastasis (M) stage, n (%)	M0	594 (32)	130 (37)	134 (34)	119 (33)	124 (32)	87 (23)
	M1	564 (30)	51 (14)	89 (23)	99 (27)	146 (37)	179 (47)
	MX	727 (39)	174 (49)	171 (43)	143 (40)	121 (31)	118 (31)
Gleason Grade Groups (GGG), n (%)	GGG1	213 (11)	48 (14)	38 (10)	48 (13)	39 (10)	40 (10)
	GGG2	222 (12)	60 (17)	45 (11)	42 (12)	41 (11)	34 (9)
	GGG3	257 (14)	63 (18)	52 (13)	54 (15)	49 (13)	39 (10)
	GGG4	380 (20)	72 (20)	85 (22)	61 (17)	85 (22)	77 (20)
	GGG5	574 (31)	60 (17)	122 (31)	115 (32)	133 (34)	144 (38)
	Missing	239 (13)	52 (15)	52 (13)	41 (11)	44 (11)	50 (13)
Prostate-specific antigen (PSA) at diagnosis (ng/mL), median (IQR)		48 (18–157)	31.5 (16–66)	49 (17–122)	39 (15–129)	57 (19–199)	104 (24–470)
PSA at diagnosis (ng/mL), n (%)	< = 10	247 (13)	45 (13)	57 (15)	56 (16)	46 (12)	43 (11)
	10–20	298 (16)	75 (21)	60 (15)	66 (18)	58 (15)	39 (10)
	20–50	409 (22)	110 (31)	82 (21)	72 (20)	84 (22)	61 (16)
	50–100	308 (16)	59 (17)	79 (20)	56 (16)	67 (17)	47 (12)
	100–500	391 (21)	47 (13)	89 (23)	66 (18)	89 (23)	100 (26)
	500+	207 (11)	12 (3)	23 (6)	37 (10)	44 (11)	91 (24)
	Missing	25 (1)	7 (2)	4 (1)	8 (2)	3 (1)	3 (1)
Age at CRPC diagnosis (years), median (IQR)		77 (70–83)	81 (76–84)	78 (71–84)	76 (69–83)	76 (70–82)	76 (67–82)
Age at CRPC diagnosis (years), n (%)	≤65	216 (12)	14 (4)	36 (9)	51 (14)	49 (13)	66 (17)
	66–70	258 (14)	24 (7)	50 (13)	54 (15)	56 (14)	74 (19)
	71–75	309 (16)	48 (14)	70 (18)	63 (18)	77 (20)	51 (13)
	76–80	405 (22)	89 (25)	84 (21)	78 (22)	85 (22)	69 (18)
	81–85	427 (23)	110 (31)	91 (23)	65 (18)	88 (23)	73 (19)
	86+	270 (14)	70 (20)	63 (16)	50 (14)	36 (9)	51 (13)
Calendar year of CRPC diagnosis, n (%)	2006–2009	598 (32)	68 (19)	117 (30)	143 (40)	132 (34)	138 (36)
	2010–2013	1131 (60)	257 (72)	244 (62)	193 (54)	223 (57)	214 (56)
	2014–2016	156 (8)	30 (9)	33 (8)	25 (7)	36 (9)	32 (8)
Time from prostate cancer diagnosis to CRPC diagnosis							
	0–6 months	62 (3)	0 (0)	1 (0)	6 (2)	17 (4)	38 (10)
	7–12 months	317 (17)	5 (1)	31 (8)	59 (16)	99 (25)	123 (32)
	1–2 years	457 (24)	47 (13)	119 (30)	103 (29)	106 (27)	82 (21)
	2–4 years	515 (27)	149 (42)	140 (36)	87 (24)	80 (21)	59 (15)
	>4 years	534 (28)	154 (43)	103 (26)	106 (29)	89 (23)	82 (21)
Charlson Comorbidity Index, n (%)	0	1280 (70)	235 (66)	262 (67)	244 (70)	268 (69)	271 (71)
	1	235 (13)	41 (12)	56 (14)	56 (16)	45 (12)	37 (10)
	2	170 (9)	39 (11)	37 (9)	26 (7)	37 (10)	31 (8)
	≥3	200 (11)	40 (11)	39 (10)	35 (10)	41 (11)	45 (12)
ATC code anatomical main group level, n (%)	A	2382	423 (13)	394 (12)	431 (13)	566 (15)	568 (14)
	B	1611	359 (11)	351 (10)	274 (8)	313 (8)	314 (8)
	C	3664	853 (26)	836 (25)	634 (19)	651 (17)	690 (17)
	D	575	132 (4)	120 (4)	103 (3)	131 (3)	89 (2)
	G	558	77 (2)	108 (3)	128 (4)	121 (3)	124 (3)
	H	334	49 (2)	45 (1)	57 (2)	89 (2)	94 (2)
	J	1216	158 (5)	203 (6)	266 (8)	258 (7)	331 (8)
	L	2483	345 (11)	458 (14)	494 (15)	578 (15)	608 (15)
	M	771	94 (3)	126 (4)	131 (4)	193 (5)	227 (6)
	N	2875	459 (14)	457 (13)	532 (16)	654 (17)	773 (19)
	P	33	5 (0)	6 (0)	11 (0)	3 (0)	8 (0)
	R	911	175 (5)	182 (5)	181 (5)	198 (5)	175 (4)
	S	519	122 (4)	114 (3)	100 (3)	77 (2)	106 (3)
	V	18	8 (0)	0 (0)	4 (0)	5 (0)	1 (0)

CRPC: Castration resistant prostate cancer; IQR: Interquartile range; ATC: Anatomical therapeutic chemical classification system.

Data on 112 drugs that were prescribed to at least 1% of men who died were used to create the DCI-CRPC. The selected drugs were not exactly the same as those used in the original DCI. Out of the 112 drugs, 16 were present only in DCI-CRPC and not in the original DCI and 10 drugs were present in the original DCI but not in DCI-CRPC (**S1 Table**).

**Fig 1:**
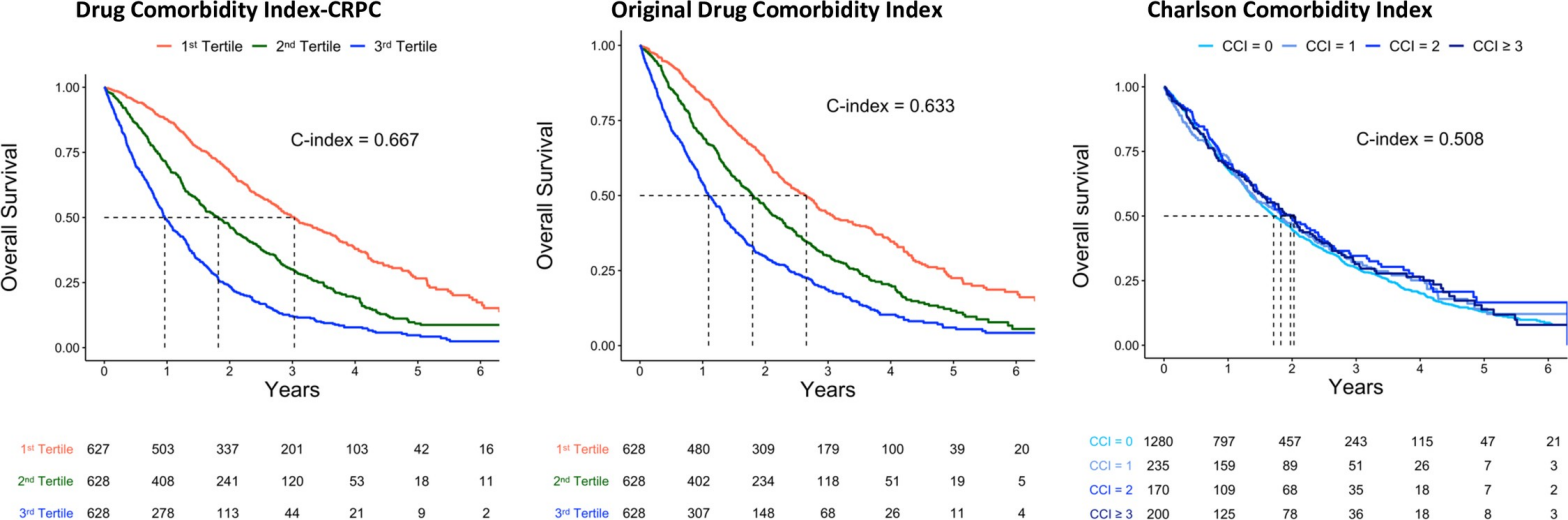
Overall survival for 1885 men with castration resistant prostate cancer (CRPC), stratified in tertiles of the Drug Comorbidity Index developed for CRPC (DCI-CRPC), the original DCI, and the Charlson Comorbidity Index.

Median DCI-CRPC was 1.5 (interquartile range 0.66–2.93). The study population was stratified according to the tertiles of this index. The majority of men had CCI = 0 in all strata of DCI, and the proportions of men according to CCI scores were similar in all DCI strata (**S2 Table**). Median overall survival was 3.0 years (95% Confidence Interval [CI] 2.8–3.4) in the first tertile of DCI-CRPC and 1.0 year (95% CI 0.9–1.1) in the third tertile (**Fig 1**).

**Fig 2:**
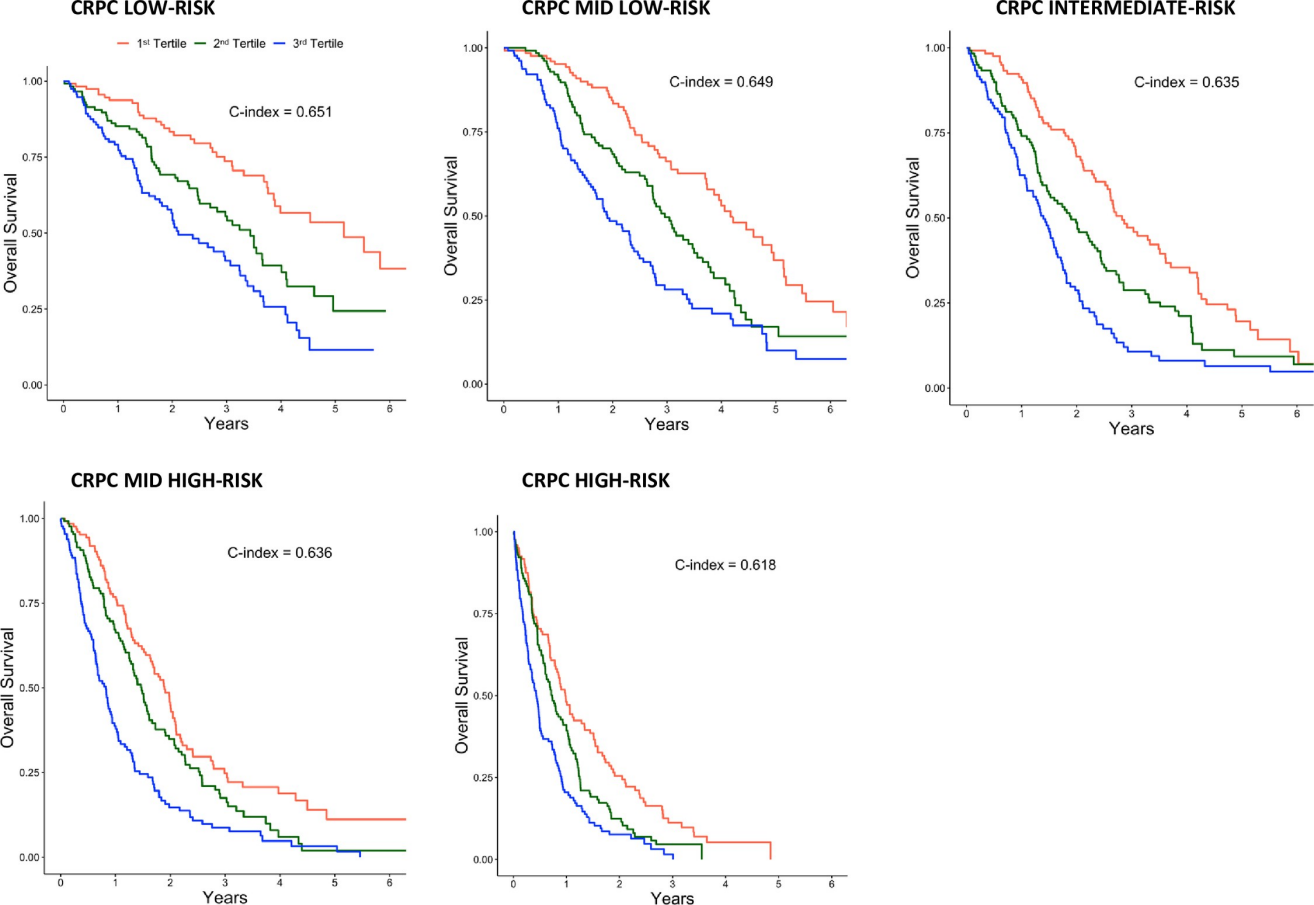
Overall survival in 1885 men with castration resistant prostate cancer (CRPC) in five risk categories and stratified in tertiles of the Drug Comorbidity Index for CRPC (DCI-CRPC).

The discriminative ability of univariable model for survival with the DCI-CRPC as predictor (C-index 0.667) was slightly higher compared to the original DCI (C-index 0.633), and substantially higher than for the CCI (C-index 0.508). When the discriminative ability of the DCI-CRPC was evaluated within each CRPC risk category, DCI-CRPC had the highest discriminative ability within the low-risk CRPC category (C-index 0.651), and the lowest discriminative ability in the high-risk CRPC category (C-index 0.618) (**Fig 2**). All C-indices were slightly lower after bootstrap resampling (**S3 Table**).

When calibration was evaluated after 1, 2, and 5 years of follow-up the DCI-CRPC appeared well calibrated for observed mortality at these time points (**S1 Fig**).

In the sensitivity analyses, when using a more detailed the ATC code level, retrieving filled prescriptions from a 180-day period, limiting the selection of drugs to drugs that were prescribed to at least 5% of men who died, or selecting only ATC codes with parameter estimate p-values ≤ 0.2 from the Wald-test, the results did not notably change from the main analysis (**S2 Fig**).

## Discussion

Our DCI predicted risk of death from all causes with higher accuracy than the Charlson Comorbidity Index (CCI). The DCI-CRPC was able to identify patient strata with notably different survival probability also within CRPC risk categories. The index was least discriminative in the highest risk category, in which prostate cancer was by far the most common cause of death.

We compared univariable models instead of optimising prediction with multivariable models that would have generated higher C-statistics but that would not have improved the targeted comparison with CCI. Despite this, DCI predicted death better than CCI. However, the C-index for DCI was modest and lowest in the highest risk category in which prostate cancer was by far the most common cause of death.

There are several reasons why a filled prescription for a drug can be associated with the risk of death. Commonly, the indication for the drug is associated with risk of death. For example, we found that bicalutamide, an anti-androgen, was associated with risk of death, likely due to that bicalutamide was combined with GnRH antagonist/agonist in men with particularly aggressive prostate cancer in order to obtain maximal androgen blockade. The use of opioids was also associated with risk and is likely to reflect advanced cancer with presence of severe pain. Thus, DCI may to some extent mirror cancer aggressiveness in addition to describing comorbidity. Angiotensin II receptors blockers and beta blockers were somewhat surprisingly associated with longer survival, likely due to some selection of men who filled such prescriptions. Emollients, corticosteroids ointments, and vitamin B supplementation and combinations thereof were associated with increased risk of death, possibly reflecting a general frailty or cancer progression among those prescribed such drugs**.**

The DCI predicted death from all causes in men with CRPC substantially better than the Charlson Comorbidity Index that is based on discharge diagnoses. Previous studies have shown that hospital discharge diagnoses underestimate the presence of comorbid conditions in the general population. For example, diabetes mellitus and hypertension, two conditions that increase the risk of death, are not always captured if the man has not been hospitalized [24,25]. In support of this view, we did not find higher CCI in men with higher DCI.

This study has several strengths, we had access to longitudinally collected data on PSA in a population-based cohort and comprehensive data obtained by linkages to several nationwide registries with known high quality [11,13,19]. Thus, we used longitudinal data on serum PSA levels before and at the time of CRPC diagnosis, and PSA kinetics have been shown to predict the risk of metastatic disease and death [26]. We used the same statistical approach for the computation of DCI that had previously been applied in previous studies, providing further support for its applicability [9,10]. Several nomograms have been constructed with the aim to predict survival in men with CRPC [27]. These nomograms are mostly based on information such as Eastern Cooperative Oncology Group performance status, serum levels of PSA, hemoglobin, and blood markers but a disadvantage of these factors is that they are rarely available in clinical cancer registers or administrative databases. On the other hand, information on filled prescriptions are easily available in these registries. Further studies are needed to assess if adding DCI in these models could improve their performance. Of note, given that information at the basis of our DCI is extracted from administrative or clinical registries, DCI can only be used in registry-based studies and not in clinical practice.

Limitations of our study include that there was no data on the presence of metastases at the date of diagnosis of CRPC, so we could not distinguish between non-metastatic and metastatic CRPC, and we also lacked data on several other prognostic factors such as location of metastases. Almost 30% of men in our study had metastatic prostate cancer already at date of diagnosis so these men had metastatic disease at the time of castration resistance. Another potential limitation is that the Swedish Prescribed Drug Register does not capture drugs administered in-hospital that could possibly improve the predictive ability of the DCI. Finally, use of drugs varies between countries and over time so our results might not be applicable to all other settings. For example, novel treatments for CRPC such as abiraterone and enzalutamide have been introduced after the study period, so the list of drugs for DCI in a contemporary cohort of men with CRPC would be different from ours. However, the original DCI that did not include cancer drugs was only marginally inferior in terms of accuracy to the tailor-made DCI-CRPC.

## Conclusion

A Drug Comorbidity Index (DCI) based on filled prescriptions predicted death in men with castration resistant prostate cancer substantially better than the Charlson Comorbidity Index. The tailor-made DCI-CRPC performed slightly better than the original DCI that was constructed on prostate cancer-free men from the general population. The discrimination of these indices was better in men with low-risk CRPC than in men with high-risk CRPC. We argue that our Drug Comorbidity Index is a useful predictor of death in register-based studies of men with castration resistant prostate cancer.

## Supporting information

S1 Fig Calibration plots after bootstrapping (1000 resamples) at 1, 2, and 5 years follow-up. (DOCX)

S2 Fig Sensitivity analysis of the model for overall survival according to DCI tertiles. (DOCX)

S1 Table ATC-code, chemical subgroup, and weights according to log hazard ratios from univariable Cox regression models predicting death. (DOCX)

S2 Table Charlson Comorbidity Index (CCI) and Drug Comorbidity Index (DCI) stratification in the study cohort. (DOCX)

S3 Table Harrell’s C-index after bootstrapping (1000 resampling). (DOCX)

## References

[1] West TA, Kiely BE, Stockler MR. Estimating scenarios for survival time in men starting systemic therapies for castration-resistant prostate cancer: A systematic review of randomised trials. Eur J Cancer. 2014;50(11):1916–24. doi: 10.1016/j.ejca.2014.04.004 24825113

[2] Albertsen PC, Moore DF, Shih W, Lin Y, Li H, Lu-Yao GL. Impact of Comorbidity on Survival Among Men With Localized Prostate Cancer. J Clin Oncol. 2011;29(10):1335–41. doi: 10.1200/JCO.2010.31.2330 21357791 PMC3084001

[3] Goyal J, Pond GR, Galsky MD, Hendricks R, Small A, Tsao C-K, et al. Association of the Charlson comorbidity index and hypertension with survival in men with metastatic castration-resistant prostate cancer. Urologic Oncol Seminars Orig Investigations. 2014;32(1):36.e27–36.e34. doi: 10.1016/j.urolonc.2013.02.015 23685020

[4] Whitney CA, Howard LE, Freedland SJ, DeHoedt AM, Amling CL, Aronson WJ, et al. Impact of age, comorbidity, and PSA doubling time on long-term competing risks for mortality among men with non-metastatic castration-resistant prostate cancer. Prostate Cancer P D. 2019;22(2):252–60. 10.1038/s41391-018-0095-0 30279582

[5] Zist A, Amir E, Ocana AF, Seruga B. Impact of comorbidity on the outcome in men with advanced prostate cancer treated with docetaxel. Radiol Oncol. 2015;49(4):402–8. doi: 10.1515/raon-2015-0038 26834528 PMC4722932

[6] Pratt NL, Kerr M, Barratt JD, Kemp-Casey A, Ellett LMK, Ramsay E, et al. The validity of the Rx-Risk Comorbidity Index using medicines mapped to the Anatomical Therapeutic Chemical (ATC) Classification System. Bmj Open. 2018;8(4):e021122. doi: 10.1136/bmjopen-2017-021122 29654048 PMC5905736

[7] Sylvestre E, Bouzillé G, Chazard E, His-Mahier C, Riou C, Cuggia M. Combining information from a clinical data warehouse and a pharmaceutical database to generate a framework to detect comorbidities in electronic health records. Bmc Med Inform Decis. 2018;18(1):9. doi: 10.1186/s12911-018-0586-x 29368609 PMC5784648

[8] Korff MV, Wagner EH, Saunders K. A chronic disease score from automated pharmacy data. J Clin Epidemiol. 1992;45(2):197–203. doi: 10.1016/0895-4356(92)90016-g 1573438

[9] Gedeborg R, Garmo H, Robinson D, Stattin P. Prescription-based prediction of baseline mortality risk among older men. Plos One. 2020;15(10):e0241439. doi: 10.1371/journal.pone.0241439 33119680 PMC7595371

[10] Gedeborg R, Sund M, Lambe M, Plym A, Fredriksson I, Syrjä J, et al. An Aggregated Comorbidity Measure Based on History of Filled Drug Prescriptions: Development and Evaluation in Two Separate Cohorts. Epidemiology. 2021;32(4):607–15. doi: 10.1097/EDE.0000000000001358 33935137

[11] Enblad AP, Bergengren O, Andrén O, Larsson A, Fall K, Johansson E, et al. PSA testing patterns in a large Swedish cohort before the implementation of organized PSA testing. Scand J Urol. 2020;54(5):1–6. 32734806 10.1080/21681805.2020.1797871

[12] Ludvigsson JF, Otterblad-Olausson P, Pettersson BU, Ekbom A. The Swedish personal identity number: possibilities and pitfalls in healthcare and medical research. Eur J Epidemiol. 2009;24(11):659–67. doi: 10.1007/s10654-009-9350-y 19504049 PMC2773709

[13] Hemelrijck MV, Wigertz A, Sandin F, Garmo H, Hellstrom K, Fransson P, et al. Cohort Profile: The National Prostate Cancer Register of Sweden and Prostate Cancer data Base Sweden 2.0. Int J Epidemiol. 2012;42(4):956–67. doi: 10.1093/ije/dys068 22561842

[14] Brooke HL, Talbäck M, Hörnblad J, Johansson LA, Ludvigsson JF, Druid H, et al. The Swedish cause of death register. Eur J Epidemiol. 2017;32(9):765–73. doi: 10.1007/s10654-017-0316-1 28983736 PMC5662659

[15] Wallerstedt SM, Wettermark B, Hoffmann M. The First Decade with the Swedish Prescribed Drug Register–A Systematic Review of the Output in the Scientific Literature. Basic Clin Pharmacol. 2016;119(5):464–9. doi: 10.1111/bcpt.12613 27112967

[16] Ludvigsson JF, Andersson E, Ekbom A, Feychting M, Kim J-L, Reuterwall C, et al. External review and validation of the Swedish national inpatient register. Bmc Public Health. 2011;11(1):450. doi: 10.1186/1471-2458-11-450 21658213 PMC3142234

[17] Paner GP, Stadler WM, Hansel DE, Montironi R, Lin DW, Amin MB. Updates in the Eighth Edition of the Tumor-Node-Metastasis Staging Classification for Urologic Cancers. Eur Urol. 2018;73(4):560–9. doi: 10.1016/j.eururo.2017.12.018 29325693

[18] Epstein JI, Egevad L, Amin MB, Delahunt B, Srigley JR, Humphrey PA, et al. The 2014 International Society of Urological Pathology (ISUP) Consensus Conference on Gleason Grading of Prostatic Carcinoma. Am J Surg Pathology. 2016;40(2):244–52. 10.1097/PAS.0000000000000530 26492179

[19] Cazzaniga W, Ventimiglia E, Alfano M, Robinson D, Lissbrant IF, Carlsson S, et al. Mini Review on the Use of Clinical Cancer Registers for Prostate Cancer: The National Prostate Cancer Register (NPCR) of Sweden. Frontiers Medicine. 2019;6:51. doi: 10.3389/fmed.2019.00051 30968024 PMC6438880

[20] Quan H, Sundararajan V, Halfon P, Fong A, Burnand B, Luthi J-C, et al. Coding Algorithms for Defining Comorbidities in ICD-9-CM and ICD-10 Administrative Data. Med Care. 2005;43(11):1130–9. doi: 10.1097/01.mlr.0000182534.19832.83 16224307

[21] Khoshkar Y, Westerberg M, Adolfson J, Bill-Axelson A, Olssonl H, Eklund M, et al. Mortality in Men with Castration Resistant Prostate Cancer–A Long-term Follow Up of a Population-based Real-world Cohort. Under Review 2020. n.d.; 10.1002/bco2.116 PMC8988790 35474724

[22] Hemelrijck MV, Folkvaljon Y, Adolfsson J, Akre O, Holmberg L, Garmo H, et al. Causes of death in men with localized prostate cancer: a nationwide, population-based study. Bju Int. 2015;117(3):507–14. doi: 10.1111/bju.13059 25604807 PMC4832314

[23] Wettermark B, Hammar N, MichaelFored C, Leimanis A, Olausson PO, Bergman U, et al. The new Swedish Prescribed Drug Register—Opportunities for pharmacoepidemiological research and experience from the first six months. Pharmacoepidem Dr S. 2007;16(7):726–35. doi: 10.1002/pds.1294 16897791

[24] Campbell SE, Campbell MK, Grimshaw JM, Walker AE. A systematic review of discharge coding accuracy. J Public Health. 2001;23(3):205–11. doi: 10.1093/pubmed/23.3.205 11585193

[25] Aronsky D, Haug PJ, Lagor C, Dean NC. Accuracy of Administrative Data for Identifying Patients With Pneumonia. Am J Med Qual. 2005;20(6):319–28. doi: 10.1177/1062860605280358 16280395

[26] Howard LE, Moreira DM, Hoedt AD, Aronson WJ, Kane CJ, Amling CL, et al. Thresholds for PSA doubling time in men with non‐metastatic castration‐resistant prostate cancer. Bju Int. 2017;120(5B):E80–6. doi: 10.1111/bju.13856 28371163 PMC5617753

[27] Soest RJ van, Efstathiou JA, Sternberg CN, Tombal B. The Natural History and Outcome Predictors of Metastatic Castration-resistant Prostate Cancer. European Urology Focus. 2016;2(5):480–7. doi: 10.1016/j.euf.2016.12.006 28723513

