# Supplementary material for: A drug comorbidity index to predict mortality in men with castration resistant prostate cancer
Source: PLoS One. 2021 Jul 28;16(7):e0255239. doi: 10.1371/journal.pone.0255239 (PMC8318265; doi:10.1371/journal.pone.0255239)
Supplement: S1 Fig — (DOCX) [file pone.0255239.s001.docx]

**S4. Supplementary Figure 1:** Calibration plots after bootstrapping (1000 resamples) at 1, 2, and 5 years follow-up

| *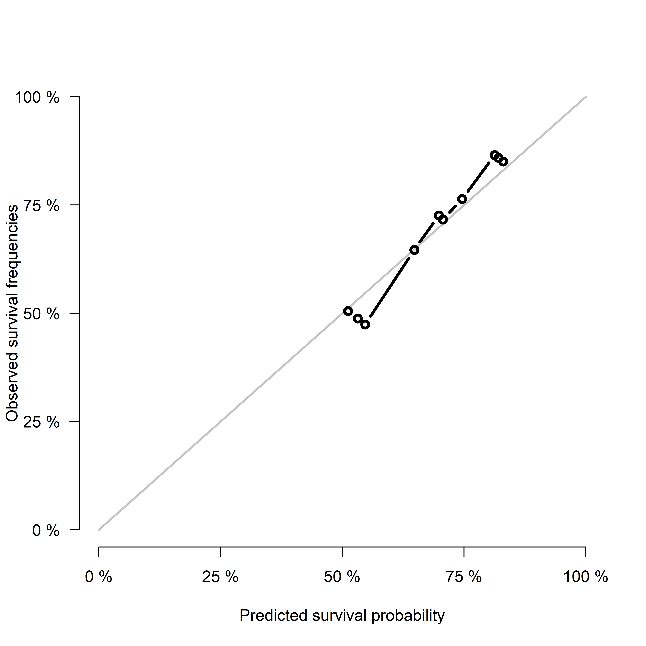* | *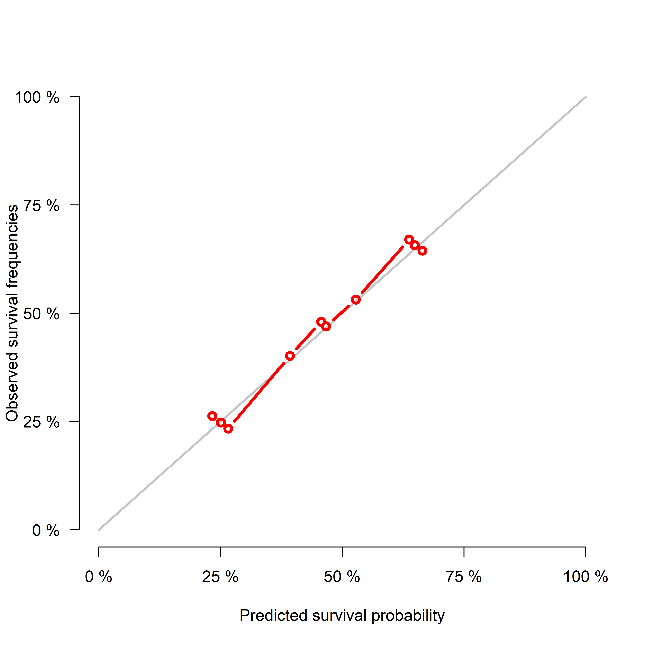* | *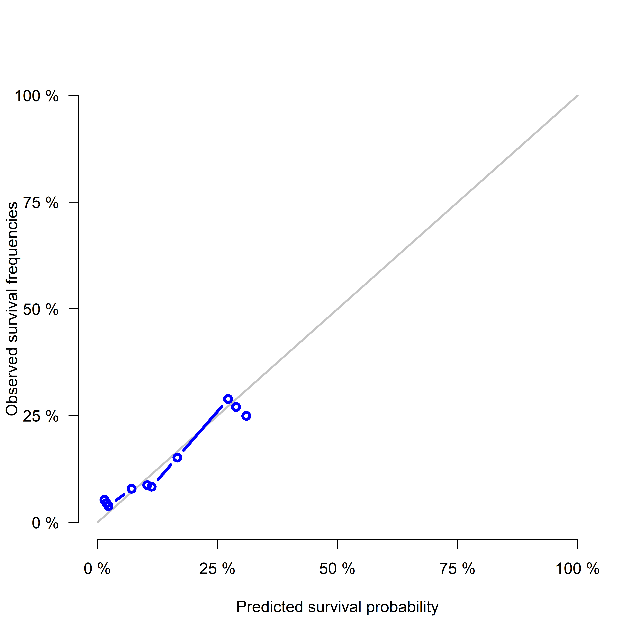* |
| --- | --- | --- |
