# Supplementary material for: A drug comorbidity index to predict mortality in men with castration resistant prostate cancer
Source: PLoS One. 2021 Jul 28;16(7):e0255239. doi: 10.1371/journal.pone.0255239 (PMC8318265; doi:10.1371/journal.pone.0255239)
Supplement: S2 Fig — (DOCX) [file pone.0255239.s002.docx]

**S5. Supplementary Figure 2:** Sensitivity analysis of the model for overall survival according to DCI tertiles

| 1. **Pharmacological subgroup level, n =75** | 1. **180 days before start of follow-up, n =99** |
| --- | --- |
| 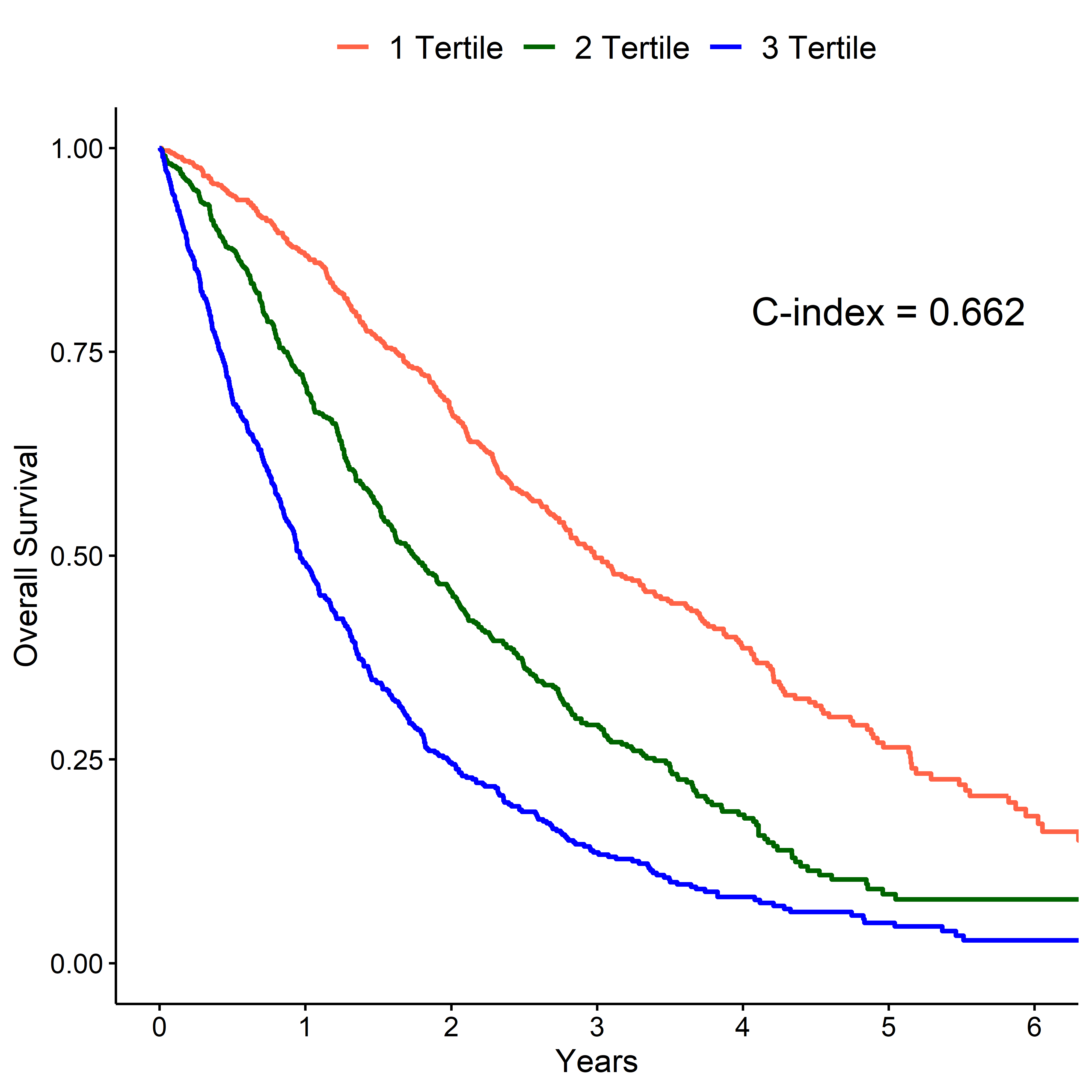 | 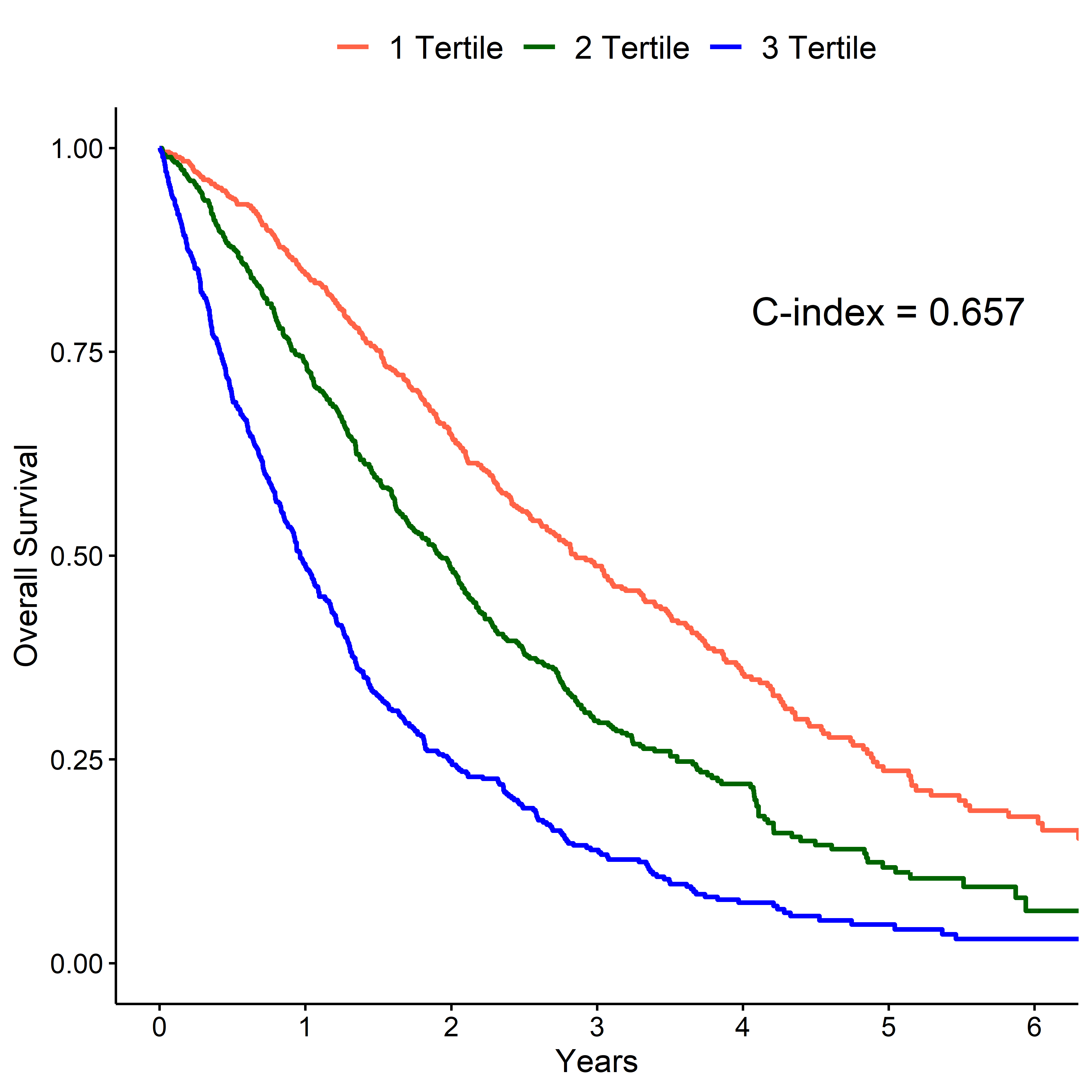 |
| 1. **Drugs contributing to 5% of death, n =46** | 1. **Wald p-value** $\boldsymbol{\leq}$**0.2, n =70** |
| 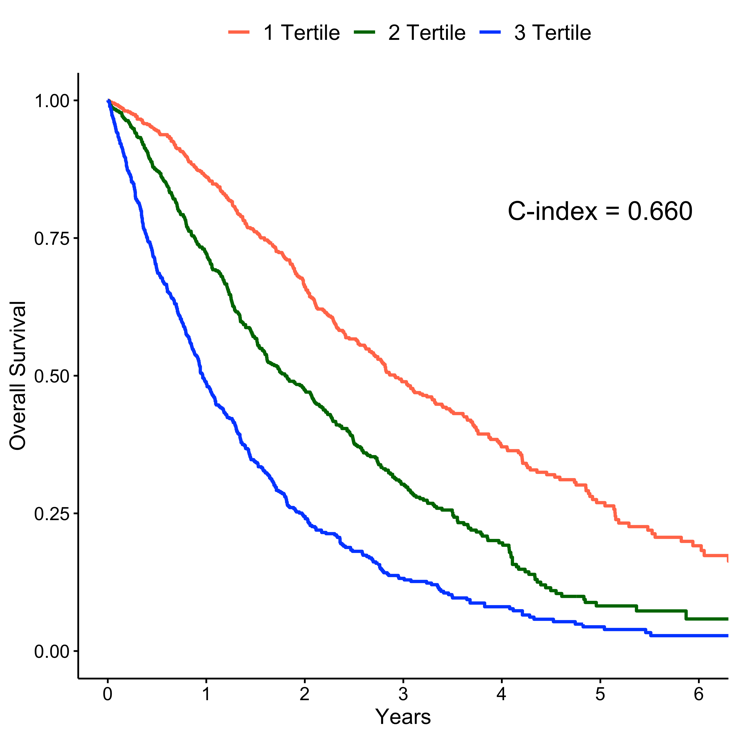 | 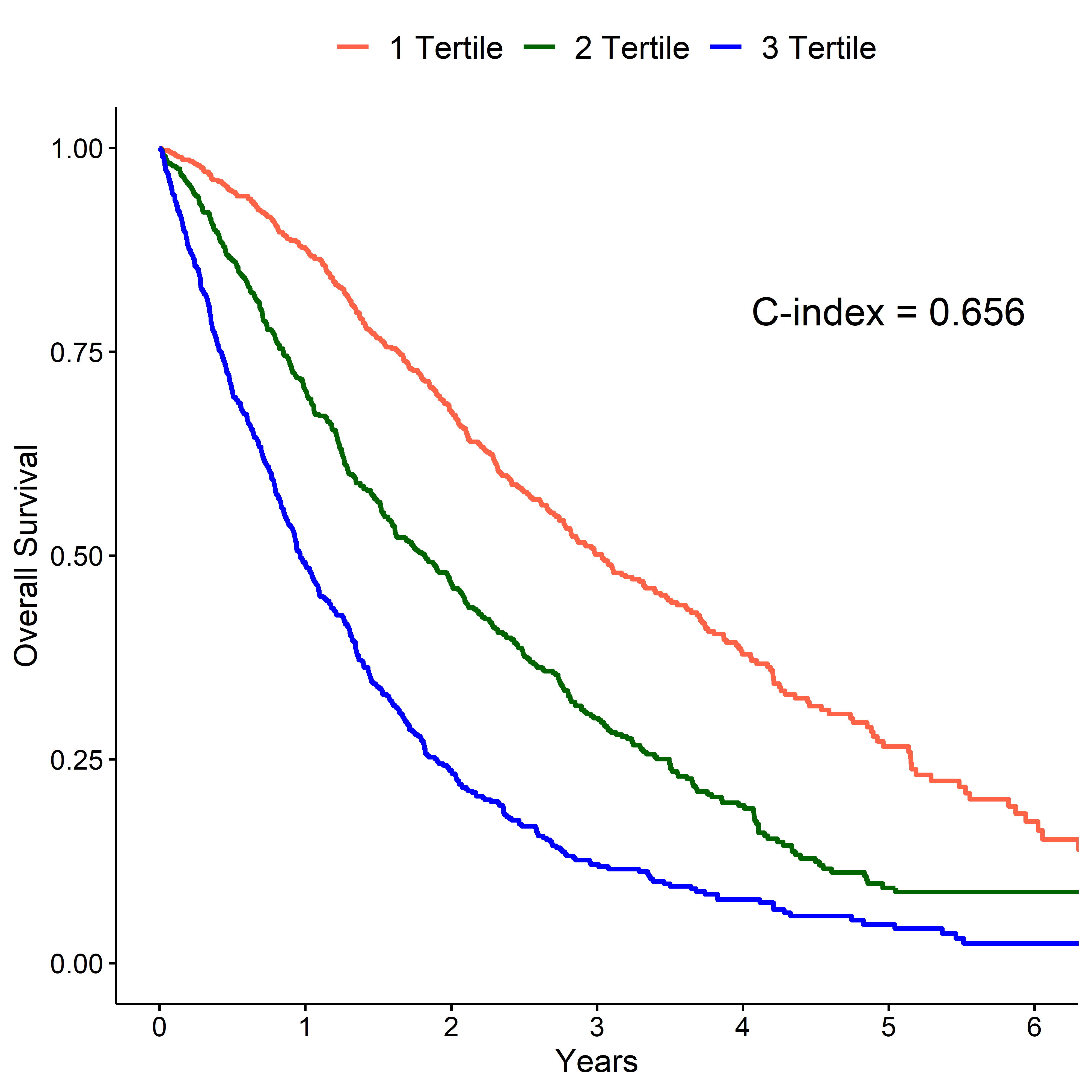 |

Sensitivity analyses for the DCI statistical model were performed by changing 1) the ATC code level, i.e. including the pharmacological subgroup level; 2) the time period before the date of start of follow-up from which the fillings for building the model were retrieved, from 365 days to 180 days; 3) limiting the analysis to drugs that were prescribed to at least 5% of men who died; 4) including only ATC codes with parameter estimate p-values $\leq$ 0.2 from the Wald-test in a Cox regression model.

DCI: Drug Comorbidity Index; ATC: Anatomical Therapeutic Chemical classification system; n: number of ATC codes used for computation of DCI
