# Supplementary material for: A drug comorbidity index to predict mortality in men with castration resistant prostate cancer
Source: PLoS One. 2021 Jul 28;16(7):e0255239. doi: 10.1371/journal.pone.0255239 (PMC8318265; doi:10.1371/journal.pone.0255239)
Supplement: S1 Table — (DOCX) [file pone.0255239.s003.docx]

**S1. Supplementary Table 1:** ATC-code, chemical subgroup, and weights according to log hazard ratios from univariable Cox regression models predicting death.

| **ATC** | **logHR** |  |
| --- | --- | --- |
| A01AA | 0,049288612 |  |
| A02BC | 0,294218452 |  |
| A03AX | 0,188387591 |  |
| A03FA | 1,055261424 |  |
| A06AB | 0,811456046 |  |
| A06AC | -0,002611905 |  |
| A06AD | 0,601705399 |  |
| A06AG | 0,57324381 |  |
| A07AA | 0,870857433 |  |
| A07DA | 0,311552894 |  |
| A10AB | -0,225189584 |  |
| A10AC | 0,018381035 |  |
| A10AD | 0,038130408 |  |
| A10AE | 0,10614471 |  |
| A10BA | 0,026649122 |  |
| A10BB | 0,109538115 |  |
| A11EA | 0,561417671 |  |
| A11EX | 1,111939577 |  |
| A12AX | 0,070585653 |  |
| A12BA | 0,049082059 |  |
| B01AA | 0,19461181 |  |
| B01AB | 0,250091597 |  |
| B01AC | 0,105259779 |  |
| B03AA | 0,579131438 |  |
| B03BA | 0,200283793 |  |
| B03BB | 0,324187648 |  |
| C01AA | 0,196059356 |  |
| C01DA | 0,07128772 |  |
| C02CA | 0,120530819 |  |
| C03AA | -0,139335649 |  |
| C03CA | 0,247270408 |  |
| C03DA | 0,270841486 |  |
| C03EA | -0,087912266 |  |
| C05AA | 0,495857202 |  |
| C07AA | 0,172017934 |  |
| C07AB | 0,097948475 |  |
| C07AG | -0,107297988 |  |
| C08CA | 0,081608392 |  |
| C09AA | -0,030004731 |  |
| C09BA | 0,068457117 |  |
| C09CA | 0,023777166 |  |
| C09DA | -0,196925623 |  |
| C10AA | -0,0145792 |  |
| D01AC | 0,153022324 |  |
| D02AE | 0,210323271 |  |
| D02AX | 0,369292657 |  |
| D07AB | 0,269010437 |  |
| D07AC | 0,163759669 |  |
| G04BD | 0,192193982 |  |
| G04BE | 0,067740677 |  |
| G04CA | -0,017154118 |  |
| G04CB | 0,501917598 |  |
| H02AB | 0,510174233 |  |
| H03AA | -0,008460077 |  |
| J01AA | 0,084760578 |  |
| J01CA | 0,512171281 |  |
| J01CE | -0,054888413 |  |
| J01CF | 0,255608036 |  |
| J01DB | 0,470897481 |  |
| J01EA | 0,456742526 |  |
| J01EE | 0,694396284 |  |
| J01FF | 0,371278348 |  |
| J01MA | 0,598731454 |  |
| J01XE | 0,341876328 |  |
| J01XX | 0,441164736 |  |
| J02AC | 1,175586294 |  |
| J05AB | 0,494165619 |  |
| L02BB | 0,47037354 |  |
| M01AB | 0,230434439 |  |
| M01AE | 0,326914139 |  |
| M01AX | -0,106104496 |  |
| M02AA | 0,219015962 |  |
| M04AA | 0,175988753 |  |
| M05BA | 0,005635488 |  |
| N01BB | 0,778020343 |  |
| N02AA | 0,660859491 |  |
| N02AB | 0,949260707 |  |
| N02AC | 0,495561266 |  |
| N02AE | 0,455869971 |  |
| N02AX | 0,597328563 |  |
| N02BE | 0,570789397 |  |
| N03AX | 0,219654786 |  |
| N04BA | 0,276468533 |  |
| N04BC | -0,050784789 |  |
| N05BA | 0,261675615 |  |
| N05BB | 0,282354165 |  |
| N05CD | 0,313021282 |  |
| N05CF | 0,337415678 |  |
| N05CM | 0,465202988 |  |
| N06AA | 0,246337508 |  |
| N06AB | 0,162874929 |  |
| N06AX | 0,221015626 |  |
| N06DA | 0,080917985 |  |
| P01AB | 0,327583061 |  |
| R01AD | -0,182408387 |  |
| R03AC | 0,155105446 |  |
| R03AK | 0,388799014 |  |
| R03BA | -0,041009676 |  |
| R03BB | 0,280383243 |  |
| R05CB | 0,246285763 |  |
| R05FA | 0,043587449 |  |
| R06AA | 0,599013596 |  |
| R06AD | 0,755957021 |  |
| R06AE | 0,727796514 |  |
| R06AX | 0,124210513 |  |
| S01AA | -0,014917861 |  |
| S01BA | -0,168005602 |  |
| S01EC | 0,223165235 |  |
| S01ED | 0,125617802 |  |
| S01EE | 0,165903546 |  |
| S01XA | 0,230362277 |  |
| S03CA | 0,288762602 |  |
| *Legend: ATC codes marked with orange are not used for calculation of DCI-original* | |  |
