# Supplementary material for: A drug comorbidity index to predict mortality in men with castration resistant prostate cancer
Source: PLoS One. 2021 Jul 28;16(7):e0255239. doi: 10.1371/journal.pone.0255239 (PMC8318265; doi:10.1371/journal.pone.0255239)
Supplement: S2 Table — (DOCX) [file pone.0255239.s004.docx]

# **S2. Supplementary Table 2:** Charlson Comorbidity Index (CCI) and Drug Comorbidity Index (DCI) stratification in the study cohort.

|  | **DCI-Q1** | **DCI-Q2** | **DCI-Q3** |
| --- | --- | --- | --- |
| **CCI = 0** | 358 **(66)** | 369 (68) | 371 (69) |
| **CCI = 1** | 59 (11) | 74 **(14)** | 69 (13) |
| **CCI ≥ 2** | 122 (23) | 96 (17) | 99 **(18)** |

*Legend: number (proportion) are shown for each strata*
