# Supplementary material for: A drug comorbidity index to predict mortality in men with castration resistant prostate cancer
Source: PLoS One. 2021 Jul 28;16(7):e0255239. doi: 10.1371/journal.pone.0255239 (PMC8318265; doi:10.1371/journal.pone.0255239)
Supplement: S3 Table — (DOCX) [file pone.0255239.s005.docx]

# **S3. Supplementary Table 3:** Harrell’s C-index after bootstrapping (1000 resampling)

|  | **C-index** | **C-index after bootstrapping** |
| --- | --- | --- |
| **Overall** | 0.667 | 0.645 |
| **CRPC low-risk category** | 0.651 | 0.624 |
| **CRPC low to mid-risk category** | 0.649 | 0.636 |
| **CRPC intermediate-risk category** | 0.635 | 0.619 |
| **CRPC mid to high-risk category** | 0.636 | 0.620 |
| **CRPC high-risk category** | 0.618 | 0.590 |
